# How postdocs benefit from building a union

**DOI:** 10.7554/eLife.05614

**Published:** 2014-11-21

**Authors:** Benjamin Cain, Jessica M Budke, Kelsey J Wood, Neal T Sweeney, Benjamin Schwessinger

**Affiliations:** Department of Physics, University of California, Davis, Davis, United States; Department of Plant Biology, University of California, Davis, Davis, United States; Genome Center, University of California, Davis, Davis, United States; Department of Molecular, Cell and Developmental Biology, University of California, Santa Cruz, Santa Cruz, United States; Department of Plant Pathology, University of California, Davis, Davis, United States and Joint Bioenergy Institute, Emeryville, United Statesbenjamin.schwessinger@gmail.com

**Keywords:** careers in science, science policy, grad school, postdoc, academic career, union, funding

## Abstract

Members of UAW 5810—the union for postdoctoral researchers at the University of California—describe how their union has led to improved terms and conditions for postdocs.

In recent years there has been an increasing amount of discussion about the problems facing the scientific research workforce in the US (Alberts et al., 2014). As the number of Ph.D. graduates has gone up (Cyranoski et al., 2011), and the competition for jobs and grants has increased, more and more young researchers are spending longer and longer in postdoctoral positions as they try to secure a permanent job (De Jesus, 2012). Consequently, the way that academia treats postdocs will have a huge impact on the future of research, in universities and beyond.

Although postdocs are essential to the academic enterprise in the US, our salaries and benefits do not reflect this. Justifications for the low pay and minimal benefits provided to postdocs usually rest on the temporary nature of postdoc appointments and the fact that employers often consider postdocs as ‘trainees’ instead of ‘staff’ (Smaglik, 2009). However, this does not reflect the true nature of our work. Postdocs drive much of the research effort in academia, while at the same time training graduate and undergraduate students, maintaining lab equipment, helping faculty apply for grants, and much more. While we do receive important training that benefits our careers (Meng and Su, 2009; Felisberti and Sear, 2014), our labor is also a crucial part of the academic workforce. All academics need to see postdocs for who we are—highly skilled, early-career researchers who play a key role in the research activities of institutions worldwide.

Postdoc demographics are also reshaping the research workforce. Recent increases in the time spent as a postdoc mean that more postdocs are starting families and trying to save for retirement at the same time as building the foundation of a productive career. On top of this, postdocs face the challenges of low wages, inadequate and expensive health insurance, and a general lack of job security. The fact that over 50% of postdocs in the US hold temporary guest worker visas that are dependent on them maintaining their current employment only exacerbates these issues.

Postdocs in the University of California (UC) system chose to address these challenges by forming the first-ever stand-alone postdoctoral researchers' union: UAW 5810. By collectively bargaining with the university, postdocs at all 10 UC campuses now have a minimum salary scale, guaranteed annual salary increases, stable and comprehensive benefits at low cost, and many other important gains (Figure 1). These have made this crucial postdoctoral time a better and more productive part of our careers.

**Figure 1.**
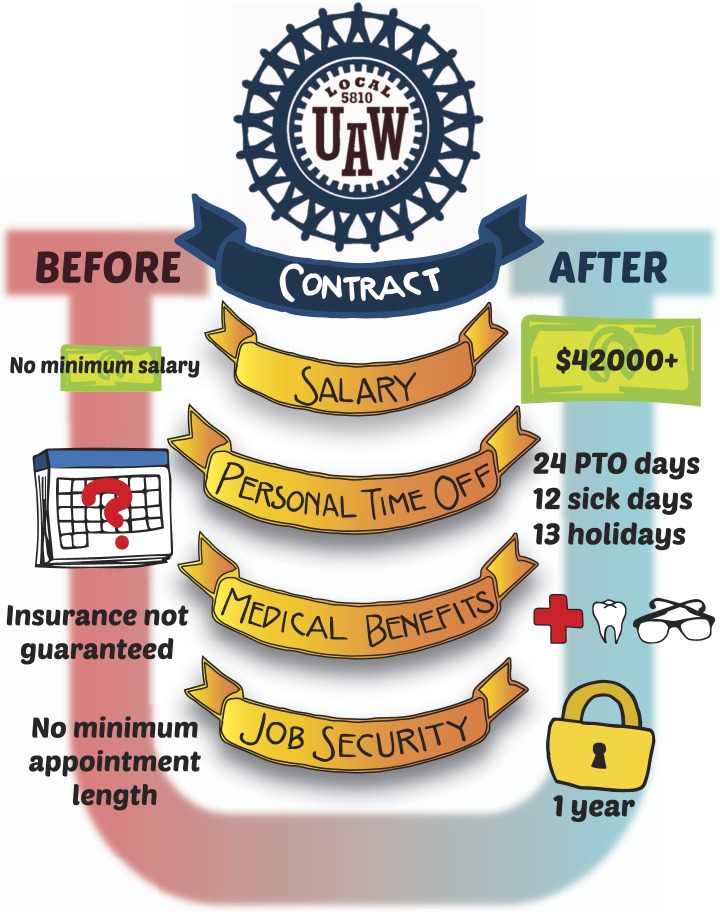
Union rights equal postdoc wins. The University of California Postdoc Union (UAW 5810) has achieved a number of contract improvements for postdocs across the university's campuses. Postdocs are now paid at least a minimum salary equal to the NIH NRSA Fellowship scale and receive guaranteed annual raises. Previously, time off was at the PI's discretion and there was no guaranteed parental/family leave. Now, contracts negotiated by UAW 5810 have increased maternity leave pay to 70% for 6 weeks. Postdocs now have 24 days personal time off (PTO) per year, in addition to 12 sick days and 13 UC ‘public’ holidays. Postdocs and dependents also receive comprehensive health, dental, and vision insurance. Postdocs must be appointed for at least 1 year; many are appointed for longer.

## A brief history of the academic labor movement in the US

The idea of a labor union for postdocs may seem odd to some, but organized labor in academia has a long history in the United States. Founded in 1916, the American Federation of Teachers (AFT) grew during the Great Depression to include university faculty unions. Albert Einstein was a charter member of AFT Local 522, the Princeton Faculty Union. On joining the union he remarked, ‘I consider it important, indeed urgently necessary, for intellectual workers to get together, both to protect their own economic status and also, generally speaking, to secure their influence in the political field’ (Einstein, 1950). With this call to action, legislation was enacted during the 1950s, 60s, and 70s to expand collective bargaining rights for higher education public employees in the US.

Faculty unionization has increased the transparency of the tenure and promotion process, provided faculty with a formalized process to address grievances, and established union contracts that include equity and non-discrimination clauses. These progressive policies may directly influence the smaller salary gap between male and female faculty and the larger representation of women faculty at unionized universities (May et al., 2010).

Over the past 45 years, US universities have shifted away from tenure-track faculty. Contingent workers, including graduate student teaching assistants, part-time/adjunct faculty, and full-time non-tenure-track faculty, now make up 75.5% of the instructional workforce (Coalition on Academic Workforce, 2012). Many of these positions do not pay a living wage and they do not offer child-care and retirement benefits: moreover, they usually only offer poor health insurance coverage (or none at all). In unionized universities, however, adjunct faculty earnings are 25% higher and access to employer-provided health and retirement benefits are better (up by 149% and 118% respectively; Coalition on Academic Workforce, 2012).

Similarly, technical and professional support staff often turn to collective bargaining to improve their working conditions. At the UC, the University Professional and Technical Employees union (UPTE-CWA 9119) overturned an arbitrary pay bonus program and won increases in base wages, among many other gains. These tangible improvements demonstrate the power of unionization to improve the working lives of academic employees.

Graduate students in the US have also unionized to improve their working conditions, starting with students at several public universities unionizing in the early 1970s. Graduate student unions have had far-reaching effects, resulting in improved wages and benefits even at non-unionized public and private universities (DeCew, 2003). These improvements also enable students from a wider range of socio-economic backgrounds to access and pursue graduate education, a key to increasing diversity in academia.

## How UAW 5810 got started

In the context of stagnant postdoc salaries and benefits, and the clear improvements won by other academics through unionization, a group of UC postdocs—many of whom were previously members of unions representing academic student employees (teaching assistants, research assistants, readers, and tutors)—approached the UAW in 2005 and asked for help forming a union. An organizing drive began and on 19 August 2008, the California Public Employment Relations Board (PERB) certified that a majority of UC postdocs had chosen UAW 5810 as their union.

Contract negotiations commenced in February 2009, but the UC administration repeatedly dragged its feet, often using the state budget crisis as an excuse, despite the fact that 85% of funding for postdoc salaries comes from federal, not state, sources. Funding for research had also more than doubled (to approximately $5bn) over the previous 15 years. On 9 June 2010 the union filed charges of unfair labor practices with PERB, citing UC's refusal to bargain in good faith after 57 bargaining sessions over a period of 17 months. Throughout this period postdocs continued their campaign for a fair contract on a number of fronts: there was informational picketing on all UC campuses; a majority of postdocs signed a public statement that was sent to the UC President, Mark Yudof; and there were direct action protests at the Office of the Chancellor on all campuses. Postdocs also overwhelmingly voted to authorize the bargaining team to call a strike if necessary.

After a majority of UC postdocs sent letters to Congress requesting action, the Education and Labor Committee of the House of Representatives held a hearing to investigate the delays. Examples of the discrimination experienced by UC postdocs were also brought up at the hearing: one postdoc testified that shortly after telling her PI that she was pregnant, she was told funding for her position was no longer available. Committee members, including Chairman George Miller, expressed shock and outrage at this treatment and at UC's delay tactics. Miller wrote to Yudof expressing ‘deep concern’, saying he ‘left the hearing thoroughly disappointed’ in UC's efforts to reach an agreement. With the combined pressure of UC postdocs and other allies (including UC students, faculty and other unions), a contract agreement was finally reached on 31 July 2010.

## Immediate improvements

With our contract came many rights, benefits, and improvements to postdoc life (Figure 1). For the first time we have a guaranteed minimum salary scale and guaranteed annual salary increases. Other benefits include equal and affordable access to health care benefits, career development rights, paid time off and sick leave, protections against discrimination, and a process for fairly resolving disputes—all major gains. And just as importantly, postdocs have an organization that is an unequivocal advocate for our interests in all areas. Postdocs come to UC to do research and pursue excellence—we deserve to be able to focus on our work, and not have to worry about adequate pay and benefits.

The economic gains for postdocs cannot be overstated. Before the union, postdocs had to negotiate individually for salary and benefits; consequently, salaries were uneven across departments and campuses and carried no guarantee of annual raises. Salaries stagnated at near-poverty levels—in one instance a UC postdoc made $18,000 per year for a full time appointment. By contrast, the union contract guarantees an experience-based minimum salary tied to a national standard (the NIH NRSA Fellowship scale). As a result, the average UC postdoc salary has increased by nearly 14% to about $47,800 (as of July 2014). Additionally, the 2014 minimum salary for a starting postdoc now exceeds the living wage for two adults in the cities near each UC campus; however, this is only true for a family of four at one campus (UC Merced). For international postdocs, higher salaries can be particularly important, as some types of guest worker visas do not allow spouses or partners to work.

Even with these advances, many postdocs and their families are still balancing present economic needs against an expectation of future academic success and a higher salary. This can easily lead some postdocs to expend personal funds, take on debt, or both, in order to make ends meet. Still, the newly won benefits, such as affordable health care that includes all dependents, are a significant achievement.

The importance of economic factors does not, however, preclude the many other benefits of unionization, such as standardizing aspects of the postdoc experience and allowing postdocs to focus on our research. For example, we now have a clear process for resolving disputes quickly and fairly, culminating in neutral third-party arbitration if necessary. Having the union as an advocate in the workplace provides incoming postdocs with a peer support system and access to the most up-to-date information on workplace rights and the means to exercise those rights. This element is again especially important for international postdocs, especially when they have to deal with something as complex as the US health care system for the first time.

By joining together collectively in a union, postdocs also gained a voice beyond the campus. For example, UAW 5810 has enabled postdocs to increase our visibility and involvement in politics that influence science and society: this is particularly important at a time when the political climate is at times regressive and ‘anti-science’. For example at the end of 2013, when the Sequester loomed and science funding in the US was threatened, we started an initiative to roll back these cuts and improve overall science funding. Thirty-nine congressional representatives from across the nation supported our initiative by signing onto a letter, circulated by Rep. George Miller and Rep. Jim McDermott, to the Congressional Leadership of both parties. Due to these and many other efforts, the deepest cuts in federal science funding have been averted, but we are continuing to push for research budget increases.

In addition, we have supported open access policies, comprehensive immigration reform, and an array of social justice causes. For instance, we co-sponsored a bill in the California State Legislature to prevent graduate student pregnancy discrimination (AB 2350), and another to extend collective bargaining rights to graduate student research assistants (AB 1834). We have also taken an interest in professional development by hosting events and panel discussions on the tenure track process, applying for fellowships, career options for Ph.Ds, open access, and immigration policies. Through this collective approach we have built a network of postdocs, former postdocs, and other members of the wider scientific community who support each other, whether it be by helping postdocs stand up for our rights in the workplace, by advocating for positive change in the political sphere, or by improving scholarly communication and the assessment of scientific achievement.

## How to build your own union

If you're interested in forming a postdoc union at your university, there are two basic steps. First, find a group of postdocs who are concerned about improving postdoc rights and working conditions, and who want to work collectively to achieve it. Starting a discussion group or organizing an advocacy campaign that supports postdocs (such as a petition to increase federal science funding) is a great way to start building a group of active and engaged colleagues. Second, contact a union that can help with the process of forming a new postdoc union.

We chose the UAW for a number of reasons. One reason was that more than 50,000 workers in higher education have joined the UAW, including postdocs at the University of Massachusetts and academic student employees at universities across the US—including graduate students at UC. In our case, choosing the union that already represented the graduate students at UC enabled us to draw on years of UAW experience in representing UC employees. The union can also help with the legal requirements for forming a new postdoc union as laws vary in different states and countries.

A key point is that while there are different types of postdoc organizations (such as postdoc associations), only a union with collective bargaining rights has the power to negotiate with the employer as equals and to reach a legally binding contract that protects the rights of all postdocs at your university. As more postdocs form unions, we will have an even stronger collective voice to advocate for improvements to postdoc working conditions and broader issues, such as increasing public investment in research and development.

As more postdocs form unions, we will have an even stronger collective voice to advocate for improvements to postdoc working conditions and broader issues.

## Looking into the future

UAW 5810 has successfully improved the lives of postdocs, and there is plenty of room for additional advances. At the end of September 2015, the first contract that we negotiated will expire, so 2015 will see UC postdocs engaging in the collective bargaining process again and working hard with the university to ensure that the postdoc experience continues to improve. UC is a world-leading research institution, and this should be reflected in how postdocs are compensated and treated.

There are certainly areas to address—for example, the minimum salary scale does not reflect the high cost of living near most UC campuses, especially in the absence of affordable childcare or housing options. A host of family-friendly benefits would reduce the economic pressure on postdoc families and make important progress towards reducing the gender inequalities that are widespread in academia. All postdocs, including international postdocs, should have the same due process rights to protect against unjust termination and to ensure we are appropriately rewarded for our work. This includes ensuring adequate recognition via journal publications and other forms of scholarly communication.

Through our union, UAW 5810, and the collective bargaining process, UC postdocs are making that happen. We have already achieved a great deal in our first few years, and we are looking forward to working with the university towards a better future for postdocs everywhere, because improvements for postdocs at UC are felt far beyond California.
